# Blended controlled-release nitrogen fertilizer increases rice post-anthesis nitrogen accumulation, translocation and nitrogen-use efficiency

**DOI:** 10.3389/fpls.2024.1354384

**Published:** 2024-04-29

**Authors:** Manman Yuan, Gang Wu, Jiabao Wang, Chuang Liu, Yegong Hu, Run Hu, Yan Zhou, Xiangming Zhang, Wenjun Wang, Yixiang Sun

**Affiliations:** ^1^ Key Laboratory of Nutrient Cycling, Resources and Environment of Anhui, Institute of Soil and Fertilizer, Anhui Academy of Agricultural Sciences, Hefei, China; ^2^ Agricultural Technology Promotion Center of Mingguang, Chuzhou, China; ^3^ Chizhou Academy of Agricultural Science, Chizhou, China; ^4^ Chaohu Agricultural Technology Promotion Center, Hefei, China

**Keywords:** economic return, yield, nitrogen translocation, post-anthesis nitrogen uptake, urea N type

## Abstract

One-time application of blended controlled-release nitrogen fertilizer (CRN) has the potential to solve the difficulty of top-dressing fertilizer in the cultivation of rice and reduce the cost of CRN fertilizer application. However, its effects on rice dry matter and nitrogen (N) accumulation and translocation, yield and N-use efficiency (NUE) remain uncertain. Field experiments were carried out at three sites (Mingguang, Chaohu, and Guichi) in the Yangtze River Delta in China to compare the effects of the conventional split applications of urea and the blended CRN and on post-anthesis dry matter and N accumulation and translocation, yield, and NUE in rice at 0, 60, 120, 180, and 240 kg N ha^-1^. The results showed that at the equal N application rates, compared under the conventional N fertilizer treatment, the blended CRN application significantly increased the rice yield by an average of 0.9-6.9%, mainly due to increase the number of spikelets per panicle. The highest yield achieved with blended CRN treatment occurred at 200 kg N ha^-1^, with an NUE of 45.9%. Moreover, in comparison to the conventional N fertilizer, the blended CRN treatment increased pre-anthesis N translocation (Pre-NT) by 1.0-19.8%, and the contribution of pre-NT to grain N by 0.2-8.7%, and NUE by 3.2-28.4%. Meanwhile, the blended CRN treatment reduced labor costs by 1800 Yuan ha^-1^ and enhanced the economic gains by 21.5-68.8%. Therefore, one-time application of blended CRN ≤ 200 kg N ha^-1^ application rate improved rice yield, NUE, and economic profit compared to equivalent rates of split applied conventional N fertilizers.

## 1 Introduction

Rice is one of the most important food crops in the world, with approximately 50% of the world’s population and more than 60% of China’s population consuming rice as their staple food (Yamaji et al., 2017; Zhang et al., 2018a). It is estimated that by 2030, China will increase rice production by 20% to meet the needs of population growth (Zhang et al., 2018a). Nitrogen (N) is one of the essential nutrients for rice growth. Growers often achieve high rice yields by increasing the application of N fertilizers. China is the largest N fertilizer user in the world, consuming more than 30% of the world’s N fertilizer (IFA, 2021). The Yangtze River Delta is one of the main rice production areas in China. The application rate of N fertilizer under farmers’ conventional practice in the whole rice season is often above 300 kg N ha^-1^. It is reported that the average rice N-use efficiency (NUE) is only 39% (Yu and Shi, 2015), which is 33.3-42.9% lower than that of the United States of America and Europe (Lassaletta et al., 2014; Yu et al., 2022). Excessive application of N fertilizer not only increases the production cost of rice, but also reduces NUE, and causes environmental problems such as soil acidification and water eutrophication (Congreves et al., 2021; Yuan et al., 2023).

Controlled-release nitrogen fertilizers (CRNs) have longer residence time in soil and are to synchronize N release with plant demand compared to conventional fertilizers. The CRNs applied at one-time can reduce 2-3 times of topdressing in rice cultivation, improve NUE, and reduce N runoff and leaching, and ammonia volatilization (Chen et al., 2020; Guo et al., 2019; Zhang et al., 2018a). The CRNs at the same N rate are more effective than the split application of urea at increasing crop yield and NUE (Lyu et al., 2021). For example, Zheng et al. (2020) reported that the rice yield and NUE of CRN-applied treatments were increased by 12.2% and 33.9%, respectively, when compared with conventional urea fertilizer treatment. Therefore, the application of CRNs to achieve simultaneous improvement of rice yield and NUE have become an important research topic.

Although CRNs increase rice yield and NUE, their agronomic and physiological mechanisms are still unclear. The grain yield of rice mainly depends on the post-anthesis accumulation of photosynthetic products and the transport and distribution efficiency of photosynthetic assimilates. It is generally believed that more than 60% of the rice grain grouting material comes from post-anthesis photosynthetic assimilates (Kumar et al., 2006; Deng et al., 2015). Moreover, the accumulation and redistribution of N metabolism and assimilation and their products in the rice vegetative and regenerating organs are also important factors affecting yield and even NUE (Cheng et al., 2010; Sun et al., 2012; Pal et al., 2017). The N uptake by rice from flowering to maturity is much lower than the total N accumulated in the grain at maturity (Mae and Ohira, 1981; Mae, 1997). About 68% of the N accumulated in vegetative organs, such as stems and leaves after flowering, are transported to the panicle for grain development (Pal et al., 2017). However, most of the above studies focused on the effect of urea on the accumulation and transport of photosynthates and N in rice (Kumar et al., 2006; Sun et al., 2012; Deng et al., 2015). The application rate, type and management of N fertilizer are known to affect rice photosynthate and N accumulation and translocation (Artacho et al., 2009; Wang et al., 2018). Therefore, it is necessary to fully understand the measure of application of CRNs and associated mechanisms to simultaneously improve rice yield and benefits.

Urea is currently the main fertilizer N source for rice. Therefore, the amount of CRNs or blended CRNs with urea applied is mainly determined by the amount of urea applied to rice (Wang et al., 2018; Lyu et al., 2021). We conducted a five-year field experiment to derive the application rate of blended CRNs for rice in the Chaohu watershed of the Yangtze River Delta in China (Yuan et al., 2023). The optimum N rate under the blended CRN treatment was determined to be 180-214 kg N ha^-1^ with one-time application, reducing the N rate by 32-64 kg N ha^-1^ compared with the conventional N fertilizer treatment. From an environmental perspective, it was elucidated that a significant reduction in ammonia volatilization in rice fields was the crucial cause for the increase in NUE under the blended CRN treatments (Yuan et al., 2023), but empirical evidence in rice agronomy and physiology is still lacking. Moreover, the data from one field site is insufficient to establish the optimal application rate of blended CRNs in the Yangtze River Delta (Yuan et al., 2023; Zhang et al., 2018b). Therefore, in this study, field experiments were conducted across three locations over two years to compare rice yield, NUE, pre-anthesis and post-anthesis biomass and N accumulation and translocation to grain yield between the blended CRN treatments and the conventional N fertilizer treatments. This study is expected to provide a physiological basis for the improvement of NUE and yield by the application of CRNs.

## 2 Materials and methods

### 2.1 Site description

**Figure 1:**
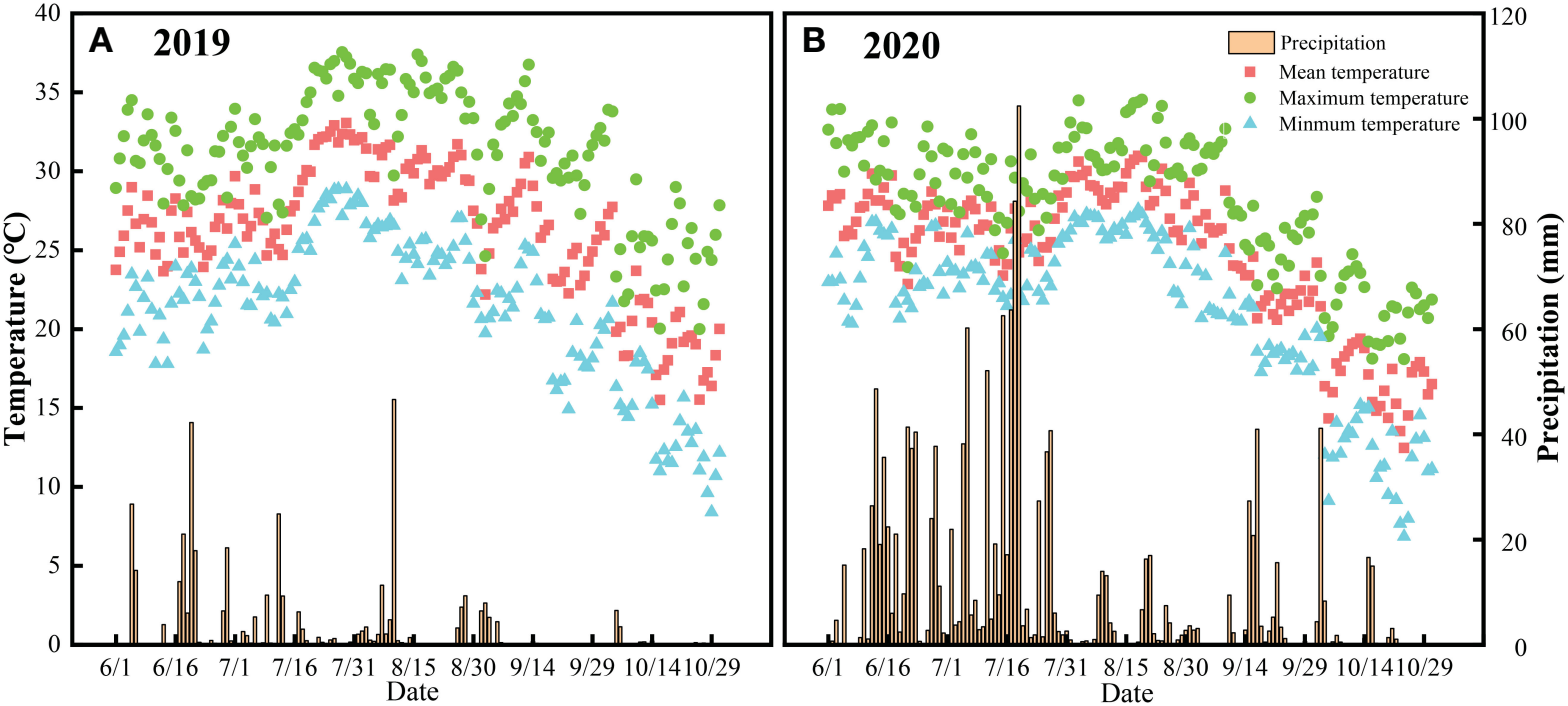
Temperature and precipitation in the rice season in 2019 **(A)** and 2020 **(B)**, respectively. The yellow bar chart represents daily precipitation, while the triangular, square, and circular point charts represent minimum, mean and maximum temperatures, respectively.

Field experiments were carried out in the 2019 and 2020 rice seasons in Mingguang, Chaohu and Guichi counties in Anhui Province, located in the Yangtze River Delta, China, which belongs to the northern subtropical monsoon climate. The annual average temperature and precipitation are 15.2°C and 953 mm in Mingguang, 16.1°C and 1030 mm in Chaohu, and 16.1°C and 1400 mm in Guichi, respectively (**Figures 1A, B**). The physical and chemical properties of the topsoil (0-20 cm) in the experimental sites are shown in **Table 1**.

**Table 1:** Basic soil properties of three experimental sites before trial establishment.

Experimental site	Longitude	Latitude	pH	Total N (g kg^-1^)	Available P (mg kg^-1^)	Available K (mg kg^-1^)	Organic matter (g kg^-1^)
Mingguang	118°01′12″ E	32°58′40″ N	6.7	0.8	18.6	97.8	10.1
Chaohu	117°46′34″ E	31°39′14″ N	7.1	1.9	9.0	198.8	29.1
Guichi	117°19′21″ E	30°31′ 04″ N	5.6	1.7	6.1	83.2	15.4

The CRNs (44.5% N) were purchased from Anhui Moith Agricultural Technology Co., Ltd. in China centered on a urea pellet coated with polyurethane. In this study, two N release periods of CRNs were used. Two CRNs with the N release periods of 40 and 90 days are abbreviated as CRN1 and CRN2, respectively. The N release characteristics of CRN1 and CRN2 in 25°C water and paddy fields for 42 and 100 days were measured in the 2019 (Yuan et al., 2023).

### 2.2 Experimental design

A split-plot design was used, with the main plot being the type of N fertilizer and its application method, and the subplot being N application rate. The numbers of main plot and subplot per replicate were 2 and 5, respectively. The plot size was 5 m × 6 m. The main plot treatments were as follows: 1. blended CRN treatment: the conventional N fertilizer, CRN1 and CRN2 with a 4:3:3 ratio of the N rate applied at one-time, and 2. conventional N fertilizer treatment: the conventional N fertilizer applied at three times as basal, tillering and panicle fertilizer. The subplot treatments were N application rates at 0, 60, 120, 180, and 240 kg N ha^-1^. The 0 rate was the same for both two main plot factors. Therefore, there were 9 treatments in the experiment. The amount and application method of N fertilizer in the different treatment are shown in **Table 2**. Triple superphosphate and potassium chloride were applied as basal fertilizers at a rate of 39 kg P ha^-1^ and 62 kg K ha^-1^, respectively.

**Table 2:** Experimental design and nitrogen fertilizer application measures.

Treatment	N application rate (kg N ha^-1^)	Type and ratio of N	N application rate in different growth stages (kg N ha^-1^)		
			Basal fertilizer	Tillering fertilizer	Panicle fertilizer
N0	0	—	—	—	—
N60	60	Urea 100%	30	18	12
N120	120	Urea 100%	60	36	24
N180	180	Urea 100%	90	54	36
N240	240	Urea 100%	120	72	48
CRN60	60	Urea: CRN1: CRN2 = 4: 3: 3	60	0	0
CRN120	120	Urea: CRN1: CRN2 = 4: 3: 3	120	0	0
CRN180	180	Urea: CRN1: CRN2 = 4: 3: 3	180	0	0
CRN240	240	Urea: CRN1: CRN2 = 4: 3: 3	240	0	0

The blended controlled-release nitrogen fertilizer (CRN) treatments are CRN60, CRN120, CRN180, CRN240. The conventional N fertilizer treatments are N60, N120, N180, N240.

The rice variety and timing of the planting and management operations in this study are shown in **Table 3**. All rice varieties used in this study were high-yield super indica rice, which are commonly grown in the region. The spacing of transplanted rice was 0.30 m (length) by 0.13 m (width).

**Table 3:** Rice varieties and planting times (month/day) of sowing, transplanting and management operations at three experiment sites.

Experimental site	Year	Rice variety	Sowing	Transplanting	Basal fertilization	Tillering topdressing	Heading topdressing	Anthesis	Harvest
Mingguang	2019	Jingliangyou huazhan	5/3	6/5	6/5	6/12	7/11	7/28	9/17
	2020	Jingliangyou huazhan	5/2	6/2	6/2	6/9/	7/8	7/25	9/13
Chaohu	2019	Chaoyou 1000	5/25	6/29	6/29	7/6	8/2	8/20	10/8
	2020	Chaoyou 1000	5/20	6/22	6/22	6/30	7/29	8/17	10/10
Guichi	2019	Jingliangyou huazhan	5/18	6/18	6/18	6/26	7/25	8/12	9/30
	2020	Jingliangyou huazhan	5/19	6/21	6/21	6/29	7/27	8/15	10/4

### 2.3 Sampling and measurements

At the anthesis and maturity, the number of tillers per unit area in each plot was measured. Based on the average number of tillers per hole, rice from three representative holes were selected for destructive sampling. The samples were divided into two parts: leaf and stem, and panicle. The panicle samples at maturity were used to determine the yield components, including the number of panicles (NP), the number of spikelets per panicle (NSP), seed-setting rate (SR), and 1000-grain weight (GW). Thereafter, the samples were dried at 105°C for 30 min and then dried continuously at 85°C for 48 h and weighed. The biomass weight was the sum of dry matter weight of shoots and panicles (Yuan et al., 2021). The dry matter samples were digested with H_2_SO_4_-H_2_O_2_. The N concentrations in the digests were determined by the Kjeldahl method (Bremner and Mulvaney, 1982).

At the final stage, the entire length of 20 rows of every plot was harvested manually to obtain the seed weight and moisture content. The rice grain yield was determined at 14% moisture content (Yang et al., 2006).

### 2.4 Calculation

The NUE was calculated according to the following equation (Sun et al., 2012):


(1) $$\begin{array}{l} \text{NUE }\left(\%\right)\;=(\text{the whole N accumulation of rice under N}-\text{application the whole Naccumulation of rice under}\\ \text{N}0)/\text{N application rate }\times 100 \end{array}$$

Based on the dry matter (DM) and N concentration, the accumulation and translocation parameters of DM and N after anthesis stage were calculated according to the following equations (Ntanos and Koutroubas, 2002; Jiang et al., 2004):


(2) $$\begin{array}{l} \text{Pre}-\text{anthesis DM translocation }\left(\text{Pre}-\text{DMT},{\text{ kg ha}}^{-1}\right)=\text{ rice panicle and plant DM accumulation}\\ \text{at anthesis stage}-\text{ rice plant DM accumulation at harvest} \end{array}$$

(3) $$\begin{array}{l} \text{Pre}-\text{anthesis DM translocation efficiency }\left(\text{Pre}-\text{DMTE},\;\%\right)=\text{ Pre}-\text{DMT}/\\ (\text{rice panicle and plant DM accumulation at anthesis stage})\;\times \;100 \end{array}$$

(4) $$\begin{array}{l} \text{Post}-\text{anthesis DM accumulation }\left(\text{Post}-\text{DMA},{\text{ kg ha}}^{-1}\right)=\text{rice grain and plant DM accumulation at}\\ \text{harvest}-\text{rice panicle and plant DM accumulation at anthesis stage} \end{array}$$

(5) $$\begin{array}{l} \text{Contribution of pre}-\text{DMT or post}-\text{DMA to grain yield }\left(\text{DMC},\;\%\right)=\text{Pre}-\text{DMT or Post}-\text{DMA}/\text{grain}\\ \text{yield}\times 100 \end{array}$$

(6) $$\begin{array}{l} \text{Pre}-\text{anthesis N translocation }\left(\text{Pre}-\text{NT},{\text{ kg ha}}^{-1}\right)=\text{rice panicle and plant N accumulation at}\\ \text{heading stage }-\text{ rice plant N accumulation at harvest} \end{array}$$

(7) $$\begin{array}{l} \text{Pre}-\text{anthesis N translocation efficiency }\left(\text{Pre}-\text{NTE},\;\%\right)=\text{Pre}-\text{NT}/(\text{rice panicle and plant N}\\ \text{accumulation at anthesis stage})\times 100 \end{array}$$

(8) $$\begin{array}{l} \text{Post}-\text{anthesis N uptake }\left(\text{Post}-{\text{NU, kg ha}}^{-1}\right)=\text{rice grain and plant N accumulation at harvest}-\\ \text{rice panicle and plant N accumulation at anthesis stage} \end{array}$$

(9) $$\begin{array}{l} \text{Contribution of pre}-\text{NT or post}-\text{NU to grain N }\left(\text{NC},\;\%\right)=\text{Pre}-\text{NT or Post}-\text{NU}/\text{rice grain N}\\ \text{accumulation at harvest }\times 100 \end{array}$$

Economic benefit calculation: the output value was calculated according to the average yield and the average price of rice sold in the 2019 and 2020 (2.5 Yuan kg^-1^). The labor cost of rice fertilization was 900 Yuan ha^-1^ each time. The fertilizer cost was calculated according to the prices and amount of fertilizer. The average prices of urea, CRN, potassium and phosphorus fertilizer were 2.0, 2.8, 2.0 and 0.6 Yuan kg^-1^ in 2019 and 2020, respectively. The other rice production costs included seed cost, irrigation cost, and disease, pest and weed management costs, etc, which were the same under all the treatments for two years. Moreover, this study mainly focused on the impact of costs associated with the type and application method of N fertilizer on the economic benefits of rice. Therefore, the net economic benefit this study was a partial net economic benefit due to the fact that the other rice production costs was not calculated.

The net economic benefit was calculated according to the following equation:


(10) Net economic benefit = Output value − Labor cost − fertilizer cost

### 2.5 Statistical analyses

Rice yield, NUE, and the accumulation and translocation parameters of dry matter and N after the anthesis stage were analyzed as split-plot analyses of variance (Equation 1), where the type and application method of N fertilizer were assigned as the main plot factor, and the N application rate as the sub-plot factor. The analyses were performed separately for each site and location, and differences in treatment means were assessed using the least significant difference test. Also, the relationships between parameters were analyzed by using the Pearson correlation method. The analyses were conducted with the SPSS 20.0 software (SPSS 22.0, IBM Corp., Armonk, NY, USA), and all analyses were reported as significant at P ≤ 0.05. The figures were created using the Origin 2022 software (OriginLab Corp., Northampton, MA, USA).

## 3 Result

### 3.1 Yield

**Figure 2:**
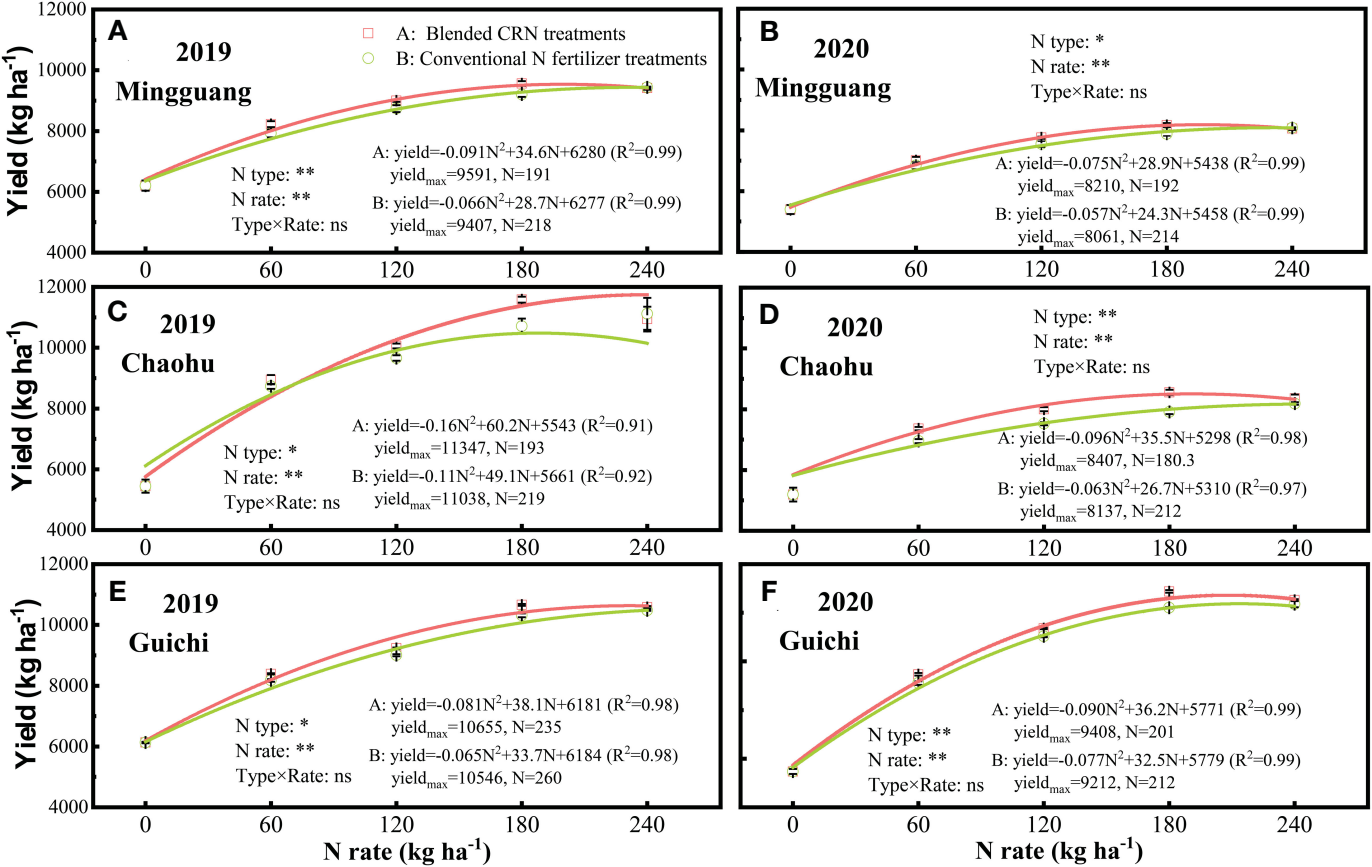
Rice yield under the treatments of blended controlled-release nitrogen fertilizer (CRN) (red square) and conventional N fertilizer treatments (green circle) applied at 0, 60, 120, 180, and 240 kg N ha^-1^ in 2019 and 2020 at Mingguang **(A, B)**, Chaohu **(C, D)** and Guichi **(E, F)** field sites, respectively. Statistically significant differences (*P<* 0.01**; *P<* 0.05*) and no significant differences (*P* > 0.05, ns) are shown. Values are the means ± SE (n=3).

Compared to the conventional N fertilizer at the equal N application rate, the blended CRN treatments increased the grain yield by an average of 0.9 - 6.7% at the three experiment sites across the two years (**Figures 2A-F**, *P<* 0.05). The rice yield increased significantly with the increase of N application rate (**Figures 2A-F**, *P<* 0.01) with no significant interaction of N type × rate (**Figures 2A-F**). The rice yield in 2020 was, on average, 18.3% lower than that in 2019. The N type had consistent effects on rice yields at the three experiment sites across the two years (**Figures 2A-F**).

The relationship between rice yield and N fertilizer rate can be simulated by a quadratic equation (**Figures 2A-F**). Under the blended CRN treatments, the highest average yield was 9604 kg ha^-1^ at 200 kg N ha^-1^ (**Figures 2A-F**). Compared with the conventional N fertilizer treatments, the maximum yield of the blended CRN treatments increased by 109-309 kg ha^-1^, with an average increase of 1.6%, and the corresponding N application rate decreasing by 10-32 kg ha^-1^, with an average decrease of 14.7% (**Figures 2A-F**).

### 3.2 Yield components

Compared with the conventional N fertilizer, the blended CRN application significantly increased the number of spikelets per panicle and seed-setting rate by averages of 1.6% and 2.0%, respectively. In contrast, the blended CRN application decreased the number of panicles by 2.0% (**Table 4**, *P*< 0.05). Increasing N application rate significantly increased these two parameters (**Table 4**, *P<* 0.01). The N type had consistent effects on rice components across the three experiment sites and two years (**Table 4**).

**Table 4:** The yield components of rice grown at Mingguang, Chaohu and Guichi field sites under the treatments of blended controlled-release nitrogen fertilizer (CRN) and conventional N fertilizer (N) applied at 0, 60, 120, 180, and 240 kg N ha^-1^ in 2019 and 2020.

Year	Treatment	2019				2020			
Location		NP (×10^4^ ha^-1^)	NSP	SR (%)	GW (g)	NP (×10^4^ ha^-1^)	NSP	SR (%)	GW (g)
Mingguang	N0	200 ± 5.5	124 ± 2.3	82.1 ± 3.3	26.1 ± 0.3	183 ± 2.5	112 ± 2.1	87.1 ± 1.5	26.0 ± 0.1
	CRN60	241 ± 2.5	136 ± 4.0	85.7 ± 1.0	27.1 ± 0.6	241 ± 2.5	129 ± 3.8	87.4 ± 1.1	26.9 ± 0.6
	CRN120	257 ± 7.4	139 ± 2.8	87.4 ± 3.3	27.3 ± 0.2	257 ± 7.4	132 ± 2.6	89.1 ± 3.3	27.1 ± 0.2
	CRN180	265 ± 4.0	146 ± 3.3	89.0 ± 1.1	27.5 ± 0.2	263 ± 3.8	139 ± 3.2	90.8 ± 1.2	26.9 ± 0.4
	CRN240	257 ± 2.4	148 ± 4.4	88.9 ± 1.9	27.5 ± 0.2	257 ± 2.4	140 ± 4.8	90.7 ± 2.0	27.1 ± 0.2
	N60	239 ± 4.0	136 ± 6.7	83.4 ± 0.7	26.7 ± 0.2	237 ± 3.2	129 ± 6.4	85.1 ± 0.7	26.5 ± 0.2
	N120	252 ± 4.4	137 ± 1.8	86.7 ± 0.8	27.1 ± 0.2	249 ± 2.7	130 ± 1.7	88.4 ± 0.8	26.9 ± 0.2
	N180	273 ± 2.0	144 ± 2.8	87.2 ± 1.2	26.8 ± 0.2	269 ± 3.4	137 ± 2.7	88.0 ± 0.7	26.4 ± 0.3
	N240	293 ± 1.1	138 ± 1.9	84.7 ± 0.8	27.2 ± 0.0	276 ± 2.4	135 ± 2.6	86.4 ± 0.8	26.6 ± 0.2
	N type	*	*	*	ns	*	*	*	ns
	N rate	**	**	ns	**	**	**	ns	*
	Type×Rate	**	ns	ns	ns	**	ns	ns	ns
Chaohu	N0	178 ± 0.7	199 ± 3.7	79.9 ± 1.1	23.6 ± 0.1	161 ± 2.6	194 ± 5.9	71.3 ± 2.1	23.2 ± 0.1
	CRN60	215 ± 2.3	220 ± 0.8	80.0 ± 1.6	23.3 ± 0.3	183 ± 5.0	224 ± 2.1	70.0 ± 0.8	23.1 ± 0.1
	CRN120	227 ± 4.1	225 ± 1.3	83.3 ± 0.9	23.4 ± 0.1	199 ± 3.2	240 ± 4.4	70.4 ± 1.0	23.3 ± 0.2
	CRN180	244 ± 2.8	250 ± 1.3	84.1 ± 2.3	23.4 ± 0.2	230 ± 4.1	241 ± 3.4	68.9 ± 1.2	23.2 ± 0.2
	CRN240	255 ± 2.6	235 ± 2.6	81.4 ± 1.8	23.2 ± 0.3	250 ± 2.1	230 ± 6.3	65.9 ± 0.9	23.0 ± 0.1
	N60	213 ± 4.8	216 ± 2.7	81.6 ± 2.7	23.4 ± 0.3	188 ± 4.6	217 ± 3.1	67.7 ± 1.0	23.4 ± 0.1
	N120	223 ± 0.6	224 ± 6.3	79.8 ± 0.1	23.7 ± 0.3	204 ± 7.8	229 ± 5.9	68.6 ± 1.3	23.0 ± 0.1
	N180	242 ± 0.4	235 ± 6.7	84.9 ± 1.4	23.8 ± 0.1	238 ± 4.8	224 ± 2.7	68.7 ± 2.5	23.2 ± 0.2
	N240	257 ± 3.7	253 ± 1.9	79.3 ± 1.0	23.1 ± 0.3	251 ± 1.1	232 ± 9.6	64.8 ± 2.7	23.2 ± 0.2
	N type	*	*	*	ns	*	*	*	ns
	N rate	**	**	ns	ns	**	**	*	ns
	Type×Rate	ns	**	ns	ns	ns	ns	ns	ns
Guichi	N0	179 ± 3.2	204 ± 2.3	89.1 ± 0.5	23.5 ± 0.0	155 ± 3.2	212 ± 0.9	80.6 ± 0.9	23.0 ± 0.2
	CRN60	198 ± 3.2	208 ± 0.7	87.9 ± 1.1	23.4 ± 0.1	190 ± 3.0	215 ± 1.9	83.0 ± 1.2	23.0 ± 0.1
	CRN120	229 ± 6.6	211 ± 1.9	83.4 ± 1.5	23.4 ± 0.1	217 ± 2.4	222 ± 1.0	80.9 ± 0.7	23.0 ± 0.1
	CRN180	255 ± 2.0	217 ± 1.0	84.0 ± 0.6	23.5 ± 0.0	249 ± 1.8	208 ± 1.1	79.3 ± 0.1	23.0 ± 0.0
	CRN240	263 ± 3.2	209 ± 1.4	84.4 ± 0.7	23.4 ± 0.2	256 ± 3.0	201 ± 2.1	76.7 ± 0.5	22.9 ± 0.1
	N60	214 ± 1.5	211 ± 1.1	88.2 ± 0.6	23.4 ± 0.1	204 ± 2.5	216 ± 1.0	81.5 ± 0.6	23.0 ± 0.1
	N120	237 ± 2.0	213 ± 1.1	83.0 ± 0.7	23.5 ± 0.0	230 ± 3.6	219 ± 1.8	79.4 ± 0.4	23.0 ± 0.2
	N180	250 ± 1.5	219 ± 1.4	82.9 ± 0.9	23.5 ± 1.0	250 ± 2.4	201 ± 2.8	75.9 ± 1.8	23.0 ± 0.1
	N240	269 ± 2.6	209 ± 0.9	82.4 ± 0.5	23.4 ± 0.0	256 ± 1.4	198 ± 1.9	75.7 ± 0.5	22.9 ± 0.1
	N type	*	*	*	ns	**	*	*	ns
	N rate	**	**	**	**	**	**	**	**
	Type×Rate	ns	ns	ns	ns	*	ns	ns	ns

Statistically significant differences (P< 0.01**; P< 0.05*) and no statistical significance (P> 0.05, ns) are shown. Values are the means ± SEs (n=3). NP, NSP, SR, GW refer to the number of panicles, number of spikelets per panicle, seed-setting rate, 1000-grain weight, respectively.

### 3.3 Nitrogen-use efficiency

**Figure 3:**
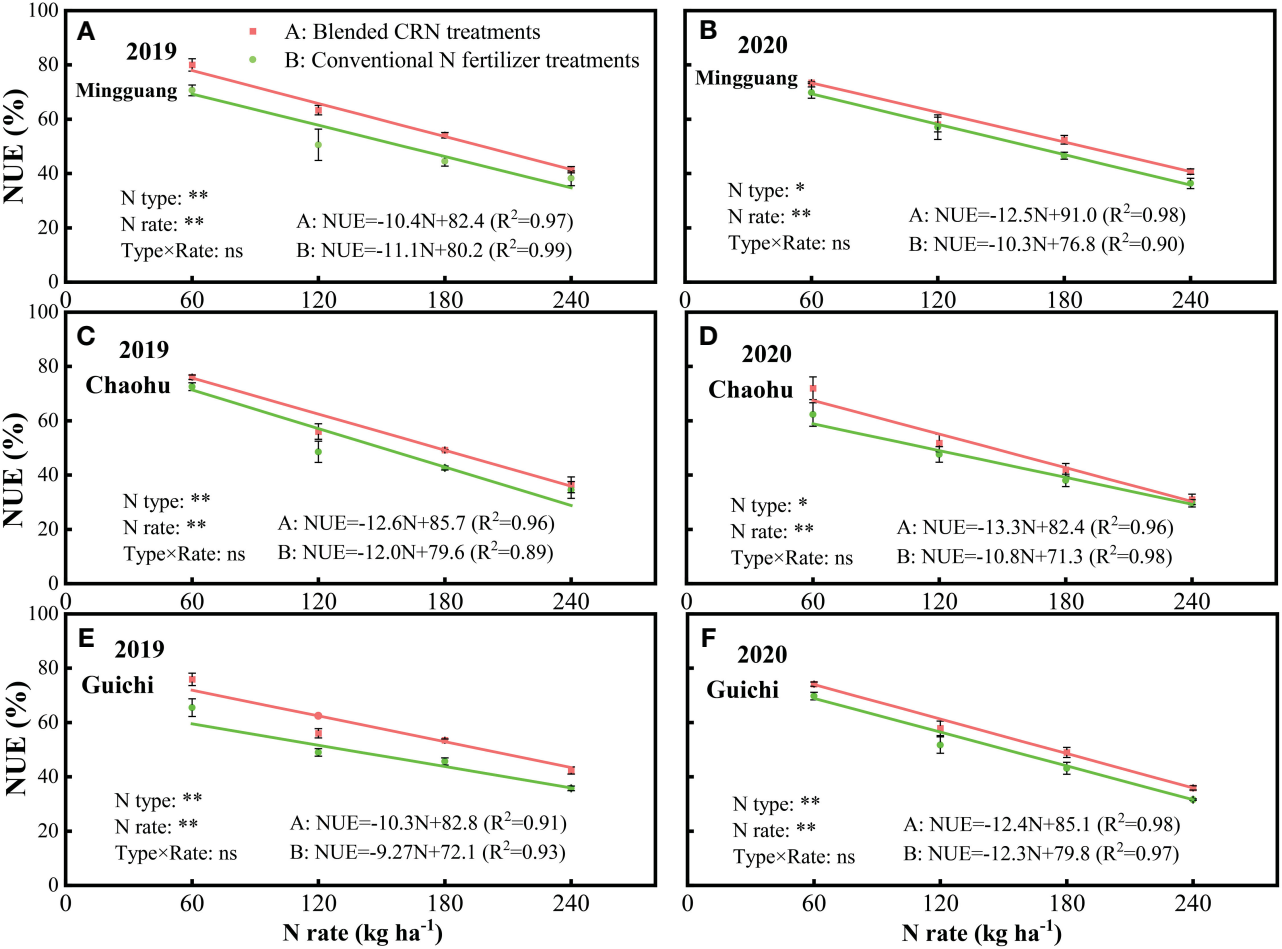
The N-use efficiency (NUE) under the treatments of blended controlled-release nitrogen fertilizer (CRN) (red) and conventional N fertilizer (green) applied at 0, 60, 120, 180, and 240 kg N ha^-1^ in 2019 and 2020 at Mingguang **(A, B)**, Chaohu **(C, D)** and Guichi **(E, F)** field sites, respective.

With the equal N application rate, NUE was significantly higher under the blended CNR than the conventional N fertilizer treatments (**Figures 3A-F**, *P<* 0.05). The increases were 5.0-16.2%, 3.2-28.4%, 10.4-21.8%, and 4.7-18.7% at 60, 120, 180 and 240 kg N ha^-1^, respectively. Increasing N application rate decreased NUE (**Figures 3A-F**, *P<* 0.01). There was no interaction between N type and rate on NUE (**Figures 3A-F**). The treatment effects on NUE followed similar trend at the three experiment sites over the two years (**Figures 3A-F**). According to the quadratic equation, the blended CNR treatments had 31.9% higher than the conventional N fertilizer treatments at the highest average grain yield.

### 3.4 Dry matter accumulation and translocation

Compared with the conventional N fertilizer, the use of blended CRN (Equation 2) significantly increased pre-anthesis DM translocation (Equation 3) (Pre-DMT), pre-anthesis N translocation efficiency (Equation 4) (Pre-DMTE), and the contribution of Pre-DMT to grain yield (Pre-DMC) by 0.8-36.7%, 0.4-25.3%, and 0.7-37.0%, respectively, but decreased the contribution (Equation 5) of pre-anthesis DM accumulation to grain yield (Post-DMC) by 0.3-25.9% (**Supplementary Table S1**; **Table 5**, *P*<0.05). Increasing N dose significantly increased Pre-DMT, Post-DMA, Pre-DMTE and Pre-DMC (**Supplementary Table S1**, *P*<0.05), but decreased Post-DMC at Chaohu and Guichi experiment sites (**Supplementary Table S1**; **Table 5**, *P<* 0.05).

**Table 5:** Pre-anthesis and post-anthesis dry matter (DM) accumulation and translocation of rice plants grown in the treatments of blended controlled-release nitrogen fertilizer (CRN) and conventional N fertilizer applied at 0, 60, 120, 180, and 240 kg N ha^-1^ in 2019 and 2020.

Year	Treatment	2019			2020		
Location		Pre-DMTE (%)	Pre-DMC (%)	Post-DMC (%)	Pre-DMTE (%)	Pre-DMC (%)	Post-DMC (%)
Mingguang	N0	26.7 ± 3.3	23.9 ± 3.5	76.1 ± 3.5	26.8 ± 1.4	23.8 ± 1.4	76.2 ± 1.4
	CRN60	28.5 ± 0.6	35.3 ± 1.1	64.7 ± 1.1	30.3 ± 4.0	37.2 ± 1.4	62.8 ± 4.9
	CRN120	24.7 ± 3.6	29.7 ± 4.0	70.3 ± 4.0	29.8 ± 3.9	37.0 ± 3.0	63.0 ± 3.0
	CRN180	23.9 ± 1.3	31.6 ± 2.1	68.4 ± 2.1	26.4 ± 1.3	35.7 ± 1.8	64.3 ± 1.8
	CRN240	23.2 ± 1.9	32.6 ± 2.9	67.4 ± 2.9	24.8 ± 2.2	33.1 ± 3.1	66.9 ± 3.1
	N60	25.1 ± 3.3	30.2 ± 4.2	69.8 ± 4.2	28.4 ± 3.5	35.2 ± 4.5	64.8 ± 4.5
	N120	23.9 ± 2.4	29.5 ± 3.1	70.5 ± 3.1	26.4 ± 0.5	32.6 ± 0.6	67.4 ± 0.6
	N180	23.6 ± 1.6	29.2 ± 2.3	70.8 ± 2.3	23.5 ± 0.5	30.6 ± 0.9	69.4 ± 0.9
	N240	22.7 ± 1.4	30.1 ± 2.3	69.9 ± 2.3	24.4 ± 0.8	32.1 ± 1.3	67.9 ± 1.3
	N type	*	*	*	*	*	*
	N rate	ns	ns	ns	ns	**	**
	Type×Rate	ns	ns	ns	ns	ns	ns
Chaohu	N0	18.1 ± 0.9	21.8 ± 0.7	78.2 ± 0.7	17.5 ± 0.2	19.0 ± 0.3	81.0 ± 0.3
	CRN60	21.2 ± 0.8	25.5 ± 1.0	74.5 ± 1.0	23.2 ± 1.8	27.1 ± 0.8	72.9 ± 0.8
	CRN120	17.9 ± 1.2	20.6 ± 1.7	79.4 ± 1.7	27.9 ± 0.9	34.7 ± 1.9	65.3 ± 1.9
	CRN180	23.7 ± 1.4	29.9 ± 1.7	70.1 ± 1.7	27.2 ± 1.1	35.8 ± 1.9	64.2 ± 1.9
	CRN240	22.5 ± 1.2	27.4 ± 0.8	72.6 ± 0.8	23.7 ± 1.7	31.9 ± 1.7	68.1 ± 1.7
	N60	17.9 ± 1.0	21.6 ± 0.2	78.4 ± 0.2	21.7 ± 2.0	26.0 ± 2.0	74.0 ± 2.0
	N120	17.6 ± 1.0	19.4 ± 0.7	80.6 ± 0.7	24.1 ± 1.4	27.8 ± 0.4	72.2 ± 0.4
	N180	23.6 ± 2.0	27.8 ± 1.1	72.2 ± 1.1	23.7 ± 1.6	30.3 ± 0.3	69.7 ± 0.3
	N240	18.9 ± 1.7	24.0 ± 2.0	76.0 ± 2.0	23.3 ± 1.1	29.8 ± 2.0	70.2 ± 2.0
	N type	*	*	*	*	*	*
	N rate	*	*	*	**	**	**
	Type×Rate	ns	ns	ns	ns	ns	ns
Guichi	N0	12.6 ± 0.5	12.8 ± 0.6	87.2 ± 0.6	12.7 ± 1.2	15.4 ± 1.1	84.6 ± 1.1
	CRN60	17.7 ± 1.3	20.8 ± 1.7	79.2 ± 1.7	21.4 ± 0.8	26.1 ± 1.1	73.9 ± 1.1
	CRN120	21.2 ± 1.1	26.4 ± 1.3	73.6 ± 1.3	31.8 ± 1.2	46.4 ± 0.9	53.6 ± 0.9
	CRN180	31.7 ± 1.9	44.0 ± 2.5	56.0 ± 2.5	38.1 ± 2.4	59.2 ± 4.4	40.8 ± 4.4
	CRN240	31.2 ± 2.5	44.1 ± 4.1	55.9 ± 4.1	36.7 ± 1.0	58.0 ± 2.0	42.0 ± 2.0
	N60	15.0 ± 1.7	15.2 ± 2.1	84.8 ± 2.1	18.9 ± 3.9	23.5 ± 6.1	76.5 ± 6.1
	N120	18.3 ± 0.6	20.5 ± 0.5	79.5 ± 0.5	27.7 ± 0.9	38.1 ± 0.7	61.9 ± 0.7
	N180	27.4 ± 2.0	33.8 ± 2.4	66.2 ± 2.4	30.4 ± 1.2	44.9 ± 1.8	55.1 ± 1.8
	N240	30.8 ± 1.2	40.5 ± 1.1	59.5 ± 1.1	33.4 ± 0.6	51.1 ± 0.6	48.9 ± 0.6
	N type	**	**	**	**	**	**
	N rate	**	**	**	**	**	**
	Type×Rate	ns	ns	ns	ns	**	**

Statistically significant differences (P< 0.01**; P< 0.05*) and no statistical significance (P >0.05, ns) are shown. Values are the means ± SEs (n=3). Pre-DMTE, Pre-DMC, and Post-DMC refer to pre-anthesis DM translocation efficiency, contribution of pre-anthesis DM to grain yield, and contribution of post-anthesis DM accumulation to grain yield, respectively.

### 3.5 N accumulation and translocation

Compared with the conventional N fertilizer (Equation 6), the blended CRN increased the pre-anthesis N translocation (Pre-NT) and the contribution of pre-NT to grain N (Equation 9) (Pre-NC) by 1.0-19.8% and 0.2-8.7%, respectively, while decreased the contribution of post-anthesis N translocation to grain N (Equation 7) (Post-NC) by 1.0-24.8% (**Supplementary Tables S2**; **Table 6**, *P<* 0.05). The fertilizer (Equation 8) type did not affect the pre-anthesis N translocation efficiency (Pre-NTE) (**Table 6**, *P* > 0.05). Increasing N rate significantly increased the Pre-NT, post-anthesis N uptake (Post-NU), and Post-NC, but decreased Pre-NTE and the contribution of pre-NT to grain N (Pre-NC) by 0.1-23.6% and 0.1-20.0%, respectively (**Supplementary Table S2**; **Table 6**, *P<* 0.05). The effects of N type and dose on N accumulation and translocation were consistent across the three experiment sites over two years (**Table 6**).

**Table 6:** Pre-anthesis and post-anthesis N accumulation and translocation of rice plants grown in the treatments of blended controlled-release nitrogen fertilizer (CRN) and conventional N fertilizer applied at 0, 60, 120, 180, and 240 kg N ha^-1^ in 2019 and 2020.

Year	Treatment	2019			2020		
Location		Pre-NTE (%)	Pre-NC (%)	Post-NC (%)	Pre-NTE (%)	Pre-NC (%)	Post-NC (%)
Mingguang	N0	64.5 ± 1.2	92.4 ± 1.2	7.6 ± 1.2	64.0 ± 0.8	91.1 ± 0.7	8.9 ± 0.7
	CRN60	62.9 ± 1.5	87.4 ± 0.3	12.6 ± 0.3	63.5 ± 1.6	88.4 ± 0.1	11.5 ± 0.1
	CRN120	58.9 ± 0.8	83.0 ± 1.2	17.0 ± 1.2	57.0 ± 0.8	83.0 ± 1.4	17.0 ± 1.4
	CRN180	55.7 ± 1.9	78.8 ± 1.4	21.2 ± 1.4	52.9 ± 1.6	79.1 ± 0.4	20.9 ± 0.4
	CRN240	49.0 ± 1.2	74.0 ± 1.8	26.0 ± 1.8	48.8 ± 1.3	72.7 ± 0.5	27.3 ± 0.5
	N60	62.3 ± 1.9	89.0 ± 0.3	11.0 ± 0.3	63.3 ± 2.0	88.6 ± 0.6	11.4 ± 0.6
	N120	60.0 ± 1.2	85.9 ± 1.1	14.1 ± 1.1	58.5 ± 0.5	83.4 ± 1.6	16.6 ± 1.6
	N180	55.4 ± 1.5	81.0 ± 1.9	19.0 ± 1.9	53.7 ± 1.4	81.1 ± 1.9	18.9 ± 1.9
	N240	49.5 ± 1.4	80.4 ± 0.7	19.6 ± 0.7	48.9 ± 1.5	75.0 ± 0.8	25.0 ± 0.8
	N type	ns	**	**	ns	*	*
	N rate	**	**	**	**	**	**
	Type×Rate	ns	ns	ns	ns	ns	ns
Chaohu	N0	65.8 ± 1.5	90.9 ± 0.1	9.4 ± 0.1	68.9 ± 0.5	91.2 ± 0.6	10.2 ± 0.6
	CRN60	62.8 ± 3.5	87.8 ± 0.5	12.2 ± 0.5	67.5 ± 1.0	88.7 ± 0.6	11.3 ± 0.6
	CRN120	65.2 ± 0.7	86.0 ± 0.5	14.0 ± 0.5	68.2 ± 0.5	85.7 ± 0.2	14.3 ± 0.2
	CRN180	63.8 ± 0.8	82.3 ± 0.4	17.7 ± 0.4	67.7 ± 0.9	82.0 ± 0.2	18.0 ± 0.2
	CRN240	63.0 ± 2.1	79.9 ± 0.6	20.1 ± 0.6	66.0 ± 1.7	80.4 ± 0.4	19.6 ± 0.4
	N60	63.2 ± 1.7	90.1 ± 0.3	9.3 ± 0.3	67.6 ± 2.2	91.1 ± 0.2	8.9 ± 0.4
	N120	65.2 ± 0.9	87.5 ± 0.7	12.5 ± 0.7	68.7 ± 1.7	88.2 ± 0.5	11.8 ± 0.5
	N180	64.7 ± 1.5	85.3 ± 0.3	14.7 ± 0.3	66.8 ± 3.7	85.6 ± 0.9	14.4 ± 0.9
	N240	63.0 ± 1.0	83.2 ± 0.8	16.8 ± 0.8	69.1 ± 1.5	83.6 ± 0.8	16.4 ± 0.8
	N type	ns	*	*	ns	**	**
	N rate	*	**	**	*	**	**
	Type×Rate	ns	ns	ns	ns	ns	ns
Guichi	N0	63.3 ± 0.7	91.3 ± 0.8	8.7 ± 0.8	60.8 ± 1.0	91.0 ± 0.9	9.0 ± 0.9
	CRN60	61.5 ± 0.4	88.2 ± 0.8	11.8 ± 0.8	58.7 ± 0.9	88.4 ± 0.5	11.6 ± 0.5
	CRN120	60.2 ± 0.4	85.9 ± 1.0	14.1 ± 1.0	56.7 ± 0.9	85.0 ± 0.4	15.0 ± 0.4
	CRN180	57.9 ± 0.7	82.8 ± 0.1	17.2 ± 0.1	55.2 ± 0.5	82.2 ± 0.6	17.8 ± 0.6
	CRN240	54.5 ± 0.4	78.7 ± 1.3	21.3 ± 1.3	53.8 ± 0.5	78.8 ± 0.2	21.2 ± 0.2
	N60	63.2 ± 0.3	89.0 ± 0.7	11.0 ± 0.7	57.4 ± 1.1	89.6 ± 0.8	10.4 ± 0.8
	N120	60.9 ± 0.2	87.7 ± 0.4	12.3 ± 0.4	56.8 ± 0.8	88.1 ± 0.5	11.9 ± 0.5
	N180	60.4 ± 0.3	85.4 ± 0.6	14.6 ± 0.6	56.7 ± 1.5	84.1 ± 0.1	15.9 ± 0.1
	N240	59.2 ± 2.0	82.3 ± 0.8	17.7 ± 0.8	54.4 ± 1.1	82.1 ± 0.2	17.9 ± 0.2
	N type	*	**	**	ns	**	**
	N rate	**	**	**	*	**	**
	Type×Rate	ns	*	*	ns	ns	ns

Statistically significant differences (P< 0.01**; P< 0.05*) and no statistical significance (P > 0.05, ns) are shown. Values are the means ± SEs (n=3). Pre-NTE, Pre-NC, and Post-NC refer to pre-anthesis N translocation efficiency, contribution of pre-anthesis N translocation to grain N, and contribution of post-anthesis N translocation to grain N, respectively.

### 3.6 Analysis of relationship

Dry matter and N accumulation, translocation, and utilization are closely related to yield and NUE. Rice yield was correlated positively with NP, NSP, Pre-DMT, Pre-DMC, Pre-NT, Post-NU, Post-NC but negatively with Pre-DMTE, Post-DMC, Pre-NTE, Pre-NC and NUE (**Table 7**, *P*<0.05). The NUE was correlated positively with Post-DMC and Pre-NC, but negatively with yield, NP, Pre-DMT, Pre-DMC, Post-NU and Post-NC (**Table 7**, *P*<0.05).

**Table 7:** Pearson corrections among yield, the number of panicles (NP), number of spikelets per panicle (NSP), seed-setting rate (SR), 1000-grain weight (GW), pre-anthesis dry matter (DM) translocation (Pre-DMT), pre-anthesis DM translocation efficiency (Pre-DMTE), post-anthesis DM accumulation (Post-MDA), contribution of pre-DMT (Pre-MDC), contribution of post-DMA (Post-MDC), pre-anthesis N translocation(Pre-NT), pre-anthesis N translocation efficiency (Pre-NTE), post-anthesis N uptake (Post-NU), contribution of pre-NT to grain N (Pre-NC) and contribution of post-NU to grain N (Post-NC) and N-use efficiency (NUE) of rice plants grown under various treatments in the 2019 and 2020 seasons (n=162).

Index	Yield	NP	NSP	SR	GW	Pre-DMT	Pre-DMTE	Post-DMA	Pre-DMC	Post-DMC	Pre-NT	Pre-NTE	Post-NU	Pre-NC	Post-NC	NUE
Yield	1															
NP	0.66**	1														
NSP	0.35**	-0.29**	1													
SR	0.11	0.29**	-0.58**	1												
GW	-0.14	0.41**	-0.91**	0.59**	1											
Pre-DMT	0.58**	0.58**	0.12	-0.07	-0.13	1										
Pre-DMTE	-0.23**	0.44**	-0.18*	-0.07	0.11	0.84**	1									
Post-DMA	0.01	0.12	-0.21**	-0.01	0.44**	-0.40**	-0.27**	1								
Pre-DMC	0.32**	0.51**	-0.07	-0.11	0.01	0.94**	0.95**	-0.36**	1							
Post-DMC	-0.32**	-0.51**	0.07	0.11	-0.01	-0.94**	-0.95**	0.36**	-1.00**	1						
Pre-NT	0.85**	0.77**	0.11	0.10	0.13	0.53**	0.34**	0.18*	0.38**	-0.38**	1					
Pre-NTE	-0.20*	-0.52**	0.44**	-0.52**	-0.42**	-0.40**	-0.28**	0.26**	-0.37**	0.37**	-0.09	1				
Post-NU	0.64**	0.81**	-0.11	0.18*	0.29**	0.53**	0.33**	0.18*	0.43**	-0.43**	0.69**	-0.53**	1			
Pre-NC	-0.53**	-0.76**	0.15	-0.16*	-0.30**	-0.51**	-0.33**	-0.14	-0.44**	0.44**	-0.54**	0.61**	-0.98**	1		
Post-NC	0.53**	0.76**	-0.15	0.16*	0.30**	0.51**	0.33**	0.14	0.44**	-0.44**	0.54**	-0.61**	0.98**	-1.00**	1	
NUE	-0.43**	-0.61**	-0.20	0.25	0.13	-0.43**	-0.23	-0.27	-0.31**	0.31**	-0.02	0.15	-0.75**	0.62**	-0.14*	1

Statistical significant correlation (P < 0.01, **; P < 0.05, *) and no statistical significant correlation (P > 0.05, ns) are shown.

### 3.7 Economic benefit

The effects of N type and application rate on economic benefits were assessed by average yields across three sites over two years (**Table 8**). The N fertilization increased fertilizer cost, economic output and net economic benefit increased by 38.5-179.0%, 33.5-67.5% and 21.5-68.8%, respectively (Equation 10). Compared with the conventional N fertilization, the blended CRN treatments reduced the labor cost by 1800 Yuan ha^-1^, but increased the fertilizer cost, economic output and net economic benefit by 4.5-9.8%, 0.2-5.2% and 8.8-15.3%, respectively. Using quadratic equation simulation, the highest net benefit was 21450 Yuan ha^-1^ under the blended CNR at an application rate of 189 kg ha^-1^ and NUE of 47.3%. In comparison, the highest net economic benefit was 19161 Yuan ha^-1^ under the conventional N fertilization at an application rate of 247 kg ha^-1^ and NUE of 31.9%. The blended CNR treatment had 11.9% higher net profit, but the applied N rate was 23.6% lower N dose and NUE was 50.4% higher NUE than the conventional N fertilization.

**Table 8:** The output values, labor and costs, and net economic benefits under the treatments of blended controlled-release nitrogen fertilizer (CRN) and conventional N fertilizer applied at 0, 60, 120, 180, and 240 kg N ha^-1^.

Treatment	Average yield	Output value	Labor cost	Fertilizer cost	Net economic benefit
	(kg ha^-1^)	(Yuan ha^-1^)	(Yuan ha^-1^)	(Yuan ha^-1^)	(Yuan ha^-1^)
N0	5766	14415	900	675	12840
CRN60	7945	19861	900	977	17984
CRN120	8787	21968	900	1279	19789
CRN180	9660	24150	900	1581	21669
CRN240	9436	23589	900	1883	20806
N60	7696	19239	2700	935	15603
N120	8498	21246	2700	1195	17350
N180	9187	22968	2700	1455	18813
N240	9413	23533	2700	1715	19118

The data are average in the 2019 and 2020 seasons.

## 4 Discussion

### 4.1 Rice yield

The final yield of rice results from the translocation of pre-anthesis stored DM and post-anthesis accumulation of photosynthetic products (Kumar et al., 2006). Appropriate N fertilizer type, rate and management can improve the photosynthetic rate and increase biomass and N accumulation and translocation, and hence yield (Artacho et al., 2009; Wang et al., 2018; Ju et al., 2021). The blended CRN treatment increased pre-anthesis DM translocation (Pre-DMT) and the contribution of Pre-DMT to grain yield (Pre-DMC) compared with the conventional N fertilizer treatment (**Supplementary Tables S1**, **Table 5**, *P*<0.05), which was consistent with the results of Wang et al. (2018). In addition, rice yield was positively correlated with Pre-DMT and Pre-DMC (**Table 7**, *P*<0.01), in agreement with the findings by Pal et al. (2017). This may be related to the fact that the blended CRN treatment decreased ineffective tillering to increase panicle number (**Table 4**), improved post-anthesis root function, especially cytokinin transport to leaves, and increased the accumulation of photosynthetic assimilates (Gu et al., 2017). The blended CRN might play a role in enhancing the sink and source sizes.

Rice yield also depends on yield components, including the number of panicles (NP), the number of spikelets per panicle (NSP), seed-setting rate (SR), and 1000-grain weight (GW). Previous studies have shown that one-time application of CRN increased NP, NSP, and SR of rice, thereby increasing yield (Guang et al., 2018; Wang et al., 2018). In this study, compared with the conventional urea use, one-time application of CRN significantly improved rice yield, mainly due to the increased NSP (**Table 4**). The positive correlation between NSP and yield in this study (**Table 5**) was consistent with previous studies (Artacho et al., 2009; Lan et al., 2021). Although the blended CRN treatment of significantly reduced the NP (**Table 4**), the contribution of the NP and NSP to rice yield is a trade-off process (Sui et al., 2013). Due to its slow N release at the tillering stage (Yuan et al., 2023), CRN decreased the NP of rice by 3.8-11.7% and increased the NSP by 11.8-21.8% during the heading stage compared to the common urea (Dun et al., 2023). Moreover, the lack of a significant N type effect on GW in this study (**Table 4**; *P*>0.05) was consistent with previous reports (Sui et al., 2013; Wang et al., 2018), suggesting that the GW is a stable trait determined by rice genotype.

### 4.2 Optimum N rate, NUE

Nitrogen fertilization is an important measure to promote sustainable crop production (Li et al., 2017). There is not a simple linear relationship between N application rate and rice yield but the one-variable quadratic can describe this relationship well (Ju et al., 2021). According to the quadratic equation, the optimal N application rate differed slightly for different rice varieties under the conventional N fertilization. The optimal N application rates for the highest yields of rice varieties HD-5 and YJ-8 under the conventional N fertilizer treatment were 255 and 261 kg ha^-1^, respectively (Ju et al., 2021). Under the blended CRN treatment, this study obtained the average optimal N application rate of 200 kg ha^-1^, which was lower than previous results. Wang et al. (2018) recommended the N application rate of 150 kg ha^-1^ for rice crop in the Yangtze River Basin of China to achieve a rice yield of approximately 7700 kg ha^-1^. This yield was comparable to the rice yield of 7820 kg ha^-1^ at the N application rate of 60 kg ha^-1^ in our study, with the yield further increasing with increasing N dose to 120 and 180 kg ha^-1^ (8540 and 9420 kg ha^-1^, respectively). The reason for such a discrepancy in yield response to N fertilization was related to the different rice varieties. Wang et al. (2018) tested indica varieties of rice, while this study used super hybrid rice varieties. At the equivalent N application rate, the yield of super hybrid rice was higher than that of indica rice (Hu et al., 2020; Zhu et al., 2020). Therefore, rice varieties and their yield potential should be considered when the N fertilizer program is formulated.

Intensive agricultural production in developing countries faces the dual challenge of ensuring sustained increases in crop yields and reducing the environmental risks of excessive fertilizer inputs (Chen et al., 2011). Changing N application methods, such as deep application and split application, or fertilizer type, has achieved simultaneous improvement in rice yield and NUE, mainly through reducing N losses such as NH_3_ volatilization, N runoff and NO_3_ leaching (Huang et al., 2019; Liu et al., 2016; Wang et al., 2018; Yuan et al., 2023). This and previous studies demonstrated that the single application of CRN improved NUE compared with the urea split application (Wang et al., 2018; Huang et al., 2019). This improved NUE was mainly due to the slow N release and longevity (40 and 90 d) of CRNs in the paddy fields.

The NUE is closely related to grain N accumulation, which could be derived via translocation of the pre-anthesis stored N and the post-anthesis uptake of soil N (Jiang et al., 2005; Wei et al., 2017). In this study, Pre-NC under the blended CRN treatment was 74.0-88.7% which was significantly higher than that under the conventional N fertilizer treatment (**Table 6**). The results implied that the blended CRN treatment was more conducive than the conventional N fertilizer treatment to enhancing the re-translocation of N from stems and leaves to grains. The increased yield and NUE under blended CRN fertilization had resulted from the increased Pre-NT (**Table 6**), improved photosynthetic rate, delayed leaf senescence, and increased post-anthesis carbon assimilation (Wu et al., 2018).

### 4.3 Economic benefit

Economic benefit is one of the important factors influencing farmers to adopt fertilizer management practices (Zhang et al., 2021). The relatively high price of CRNs limits their use in farming practices such as rice production (Naz and Sulaiman, 2016). However, this study suggested that the blended CRN treatments could improve the economic benefits for rice cultivation (**Table 8**). With accelerated urbanization, labor for agricultural cultivation has become expensive (Chen et al., 2014). Compared with the conventional N fertilization, the use of blended CRN could save labor costs. Under the blended CRN treatment, labor cost saving offset the increased fertilizer cost, which ultimately increased the profitability for the rice growers (**Table 8**). It appears to be an effective way to achieve high grain yield and NUE by optimizing the N management mode of CRN and urea blended application (Guo et al., 2019; Huang et al., 2019; Zhang et al., 2021).

## 5 Conclusions

At the equivalent N rate, the rice yield, NUE and economic benefit significantly increased under the blended CRN treatment. The quadratic equation estimated that the N rate for the highest yield under the blended CRN treatment was 200 kg ha^-1^ and NUE was 45.9%, which were 14.7% less and 31.9% more than those under the conventional N fertilizer treatment, respectively. The rice yield at the highest economic benefit was slightly below the highest yield. At the highest economic benefit under the blended CRN treatment, the N rate was 189 kg ha^-1^ and NUE was 47.3%, which were 23.6% less and 50.4% more than those under the conventional N fertilizer treatment, respectively. The increase in NUE was due to the increase in Pre-NT and Post-NC under the blended CRN fertilizer treatment. The increase in economic benefit was mainly due to the reduction in labor cost under the blended CRN fertilizer treatment. The application rate of the blended CRN at 189 kg N ha^-1^ is recommended for rice production in the Yangtze River Delta.

## Data availability statement

The original contributions presented in the study are included in the article/**Supplementary Material**. Further inquiries can be directed to the corresponding authors.

## Author contributions

MY: Data curation, Formal analysis, Investigation, Writing – original draft, Writing – review & editing, Conceptualization. GW: Formal analysis, Investigation, Writing – review & editing, Supervision. JW: Investigation, Writing – review & editing, Methodology, Project administration. CL: Software, Writing – review & editing, Formal analysis. YH: Resources, Writing – review & editing. RH: Resources, Writing – review & editing. YZ: Resources, Writing – review & editing. XZ: Investigation, Writing – review & editing. WW: Investigation, Writing – review & editing. YS: Conceptualization, Funding acquisition, Project administration, Writing – review & editing.

## Supplementary material

Supplementary Table 1 Pre-anthesis dry matter translocation (Pre-DMT) and post-anthesis dry matter accumulation (Post-DMA) of rice crop grown in the treatments of blended controlled-release nitrogen fertilizer (CRN) and conventional N fertilizer applied at 0, 60, 120, 180, and 240 kg N ha^-1^ in 2019 and 2020. Statistically significant differences (*P<* 0.01, **; *P<* 0.05, *) and no statistical significance (*P* > 0.05, ns) are shown. Values are the means ± SEs (n=3).

Supplementary Table 2 Pre-anthesis N translocation (Pre-NT) and post-anthesis N uptake (Post-NU) of rice grown in the treatments of blended controlled-release nitrogen fertilizer (CRN) and conventional N fertilizer applied at 0, 60, 120, 180, and 240 kg N ha^-1^ in 2019 and 2020. Statistically significant differences (*P<* 0.01, **; *P<* 0.05, *) and no statistical significance (*P* > 0.05, ns) are shown. Values are the means ± SEs (n=3).
