# Supplementary material for: Blended controlled-release nitrogen fertilizer increases rice post-anthesis nitrogen accumulation, translocation and nitrogen-use efficiency
Source: Front Plant Sci. 2024 Apr 29;15:1354384. doi: 10.3389/fpls.2024.1354384 (PMC11089134; doi:10.3389/fpls.2024.1354384)
Supplement: Supplementary Table 1 — Pre-anthesis dry matter translocation (Pre-DMT) and post-anthesis dry matter accumulation (Post-DMA) of rice crop grown in the treatments of blended controlled-release nitrogen fertilizer (CRN) and conventional N fertilizer applied at 0, 60, 120, 180, and 240 kg N ha-1 in 2019 and 2020. Statistically significant differences (P< 0.01, **; P< 0.05, *) and no statistical significance (P > 0.05, ns) are shown. Values are the means ± SEs (n=3). [file Presentation_1.pdf]

Blended controlled-release nitrogen fertilizer increases rice post-anthesis nitrogen accumulation, translocation and nitrogen-use efficiency

ManmanYuan[1], GangWu[1], Jiabao Wang[1], Yegong Hu[2], Run Hu[3], Yan Zhou[4], Chuang Liu[1], Xiangming Zhang[1], Wenjun Wang[1], Yixiang Sun[1]*

[1]Key Laboratory of Nutrient Cycling, Resources and Environment of Anhui, Institute of Soil and Fertilizer, Anhui Academy of Agricultural Sciences, Hefei 230031, China, [2]Agricultural Technology Promotion Center of Mingguang, Chuzhou 239499, China, [3]Chizhou Academy of Agricultural Sciences, Chizhou 247099, China,4Chaohu Agricultral Technology Promotion Center, Hefei, China

Correspondence: Yixiang Sun, e-mail:sunyixiang@126.com

Table S1 Pre-anthesis dry matter translocation (Pre-DMT) and post-anthesis dry matter accumulation (Post-DMA) of rice crop grown in the treatments of blended controlled-release nitrogen fertilizer (CRN) and conventional N fertilizer applied at 0, 60, 120, 180, and 240 kg N ha^{-1} in 2019 and 2020. Statistically significant differences (*P* < 0.01, **; *P* < 0.05, *) and no statistical significance (*P* > 0.05, ns) are shown. Values are the means ± SEs (n=3).

Year<br>Location	Treatment	2019 Pre-DMT (kg ha^{-1})	2019 Post-DMA (kg ha^{-1})	2020 Pre-DMT (kg ha^{-1})	2020 Post-DMA (kg ha^{-1})
Mingguang	N0	1484±229	4720±220	1282±61	4113±165
	CRN60	2808±111	5742±78	2600±310	4405±401
	CRN120	2678±463	6322±426	2791±394	5223±359
	CRN180	2909±196	6774±217	2919±124	5253±186
	CRN240	3077±262	6578±293	2668±265	5389±223
	N60	2484±379	5114±249	2399±270	4442±383
	N120	2585±304	6154±206	2536±78	4757±23
	N180	2787±222	6294±211	2410±79	5470±70
	N240	2832±221	6359±201	2596±111	5490±105
	N type	*	*	*	ns
	N rate	**	**	**	**
	Type×Rate	ns	ns	ns	ns
Chaohu	N0	1187±73	4256±146	983±36	4206±228
	CRN60	2277±120	6671±256	1912±96	5450±40
	CRN120	1955±147	8103±88	2774±89	5224±43
	CRN180	2872±308	8360±257	3059±378	5490±384
	CRN240	3226±349	8353±578	2666±142	5689±237
	N60	1622±169	5946±228	1771±399	4575±467
	N120	1939±47	7484±97	2050±49	5283±75
	N180	2590±311	7520±190	2374±163	5451±113
	N240	3222±201	7510±396	2407±63	5688±22
	N type	*	*	*	ns
	N rate	**	**	**	**
	Type×Rate	ns	ns	ns	ns
Guichi	N0	850±46	5280±111	981±10	4785±88
	CRN60	1703±80	6469±395	2244±64	4818±176
	CRN120	2381±156	6976±112	4478±193	6346±143
	CRN180	4695±187	7692±110	6202±180	5789±67
	CRN240	4664±81	7998±69	5968±160	5491±163
	N60	1276±19	6482±327	1971±176	4545±266
	N120	1888±57	6689±218	3603±34	5441±60
	N180	3489±100	7688±229	4536±35	5220±124
	N240	4241±213	7873±200	5212±168	4820±280
	N type	**	*	**	**
	N rate	**	**	**	**
	Type×Rate	**	ns	**	ns

Table S2 Pre-anthesis N translocation (Pre-NT) and post-anthesis N uptake (Post-NU) of rice grown in the treatments of blended controlled-release nitrogen fertilizer (CRN) and conventional N fertilizer applied at 0, 60, 120, 180, and 240 kg N ha^{-1} in 2019 and 2020. Statistically significant differences (P < 0.01, **; P < 0.05, *) and no statistical significance (P > 0.05, ns) are shown. Values are the means ± SEs (n=3).

Year / Location	Treatment	2019 Pre-NT (kg ha^{-1})	2019 Post-NU (kg ha^{-1})	2020 Pre-NT (kg ha^{-1})	2020 Post-NU (kg ha^{-1})
Mingguang	N0	60.7±0.5	5.0±0.8	54.7±0.7	5.4±0.5
	CRN60	87.0±1.8	10.2±0.1	79.1±1.9	10.3±0.4
	CRN120	94.1±0.7	17.0±1.6	84.1±0.8	14.3±1.6
	CRN180	97.1±3.2	23.7±1.9	88.0±2.2	20.7±0.9
	CRN240	84.9±1.8	27.3±2.8	80.7±2.4	26.2±1.1
	N60	81.9±2.1	10.1±0.6	77.7±2.3	10.0±0.6
	N120	87.2±1.0	14.3±1.5	83.3±3.1	17.0±1.5
	N180	87.8±1.6	20.7±2.4	83.2±1.5	19.5±2.1
	N240	84.2±1.2	20.5±0.8	74.8±1.1	24.9±0.8
	N type	**	*	*	ns
	N rate	**	**	**	**
	Type×Rate	ns	ns	ns	ns
Chaohu	N0	50.8±2.2	5.1±0.7	42.8±1.1	4.9±0.6
	CRN60	77.3±2.2	8.5±1.1	73.1±1.5	7.0±0.4
	CRN120	91.6±1.8	12.6±0.9	83.6±1.7	11.7±0.8
	CRN180	99.2±1.1	19.0±0.4	88.4±0.9	17.0±0.9
	CRN240	95.6±1.5	21.6±2.1	85.9±0.5	17.2±0.3
	N60	66.9±1.5	7.4±0.8	61.0±0.4	5.8±0.7
	N120	81.5±1.3	11.7±0.3	76.9±0.7	10.3±0.6
	N180	91.6±1.5	15.9±0.8	82.4±0.9	13.9±0.1
	N240	90.7±2.3	18.4±0.9	85.3±1.2	16.7±0.4
	N type	*	*	**	**
	N rate	**	**	**	**
	Type×Rate	ns	ns	ns	ns
Guichi	N0	51.3±0.6	4.9±0.1	48.7±1.2	4.8±0.3
	CRN60	76.8±3.4	8.1±1.3	71.4±1.3	8.3±0.5
	CRN120	86.0±0.7	11.7±0.5	81.6±2.0	12.1±0.6
	CRN180	97.1±1.7	17.7±0.3	87.5±0.3	16.5±0.3
	CRN240	91.0±1.8	22.1±0.5	82.4±2.7	19.6±0.6
	N60	75.5±2.2	9.4±0.4	68.4±2.8	8.0±0.2
	N120	82.2±0.6	11.6±0.7	77.8±3.0	10.6±0.9
	N180	92.3±0.5	15.8±0.3	83.1±3.1	15.8±1.6
	N240	88.7±1.8	19.1±1.4	77.4±1.4	16.9±0.8
	N type	*	ns	**	*
	N rate	**	**	**	**
	Type×Rate	ns	*	ns	ns
